# Medicine as a Calling

**Published:** 1891-09-12

**Authors:** 


### THE DOCTOR'S ARMCHAIR.

### MEDICINE AS A CALLING.

Most men have to do something in life. In youth the question is—What? Every young man should, if possible, make a point of selecting an occupation for which he has a decided preference. Because, if a man's life is to be made happy at all, the principal happiness of it must come from his work. Work is medicine; it is relief from care; it is balm in sorrow; it is expansion to the intellect and strength to the character.

The average man is what his work makes him. There are very few who have any important personality, or power, or influence, outside their work. The man tends to be submerged in the specialist, in the doctor, the lawyer, the parson, the politician, the journalist, the trader. If the doctor or the lawyer be poor at his own business, he is almost invariably an insignificant creature all round. It is of fundamental importance, therefore, that a boy be put to a calling into which he can throw the whole of his heart, soul, and energy.

Some people choose an occupation because it is respectable, because it is a gentleman's profession. This is supremely stupid. Granted that the world is conventionally divided into occupations followed by gentlemen on the one hand, and by doubtfuls on the other, must we for ever be bound by the often ridiculous conventionalities which have come down to us from past generations? What a miserable coward a man must be who is afraid to be a builder or a retail trader, lest he should not be considered a gentleman! It is a thousand times better to be a practical and successful grocer or bookseller, than an unpractical, characterless, and useless doctor or parson. The very young man makes sharp distinctions, for the most foolish and inconsequent reasons, between one class of persons and another. The man who has had experience of the world has long learnt to know better.

The doctor's calling looks very different inside from what it does outside. Probably most callings resemble it in this respect. A great many medical men refuse on any condition to put their sons into medicine. They have found the profession an occupation of unceasing, harassing, and responsible work, with poor pay. It must be admitted that the general practitioner in a third-rate neighbourhood has but a melancholy time of it. An eight hours working day would be almost heaven for him. A sixteen hours day is a very common experience. Many a doctor who works sixteen hours a-day, and has two or three disturbed nights a-week into the bargain, nevertheless soon learns to think himself very lucky if not more than twenty-five per cent. of his hard earnings is lost in bad debts. The business man would shake his head at such a state of things. It is, perhaps, happy for the doctor, and certainly for his patients, that he is seldom a keen man of business. If he were, many a poor artisan and small tradesman would make acquaintance with the debtor's court, who is now allowed to go scot free.

But though medicine has its clouded summers and its winters of discontent, its sky is not always and everywhere sunless. There are prizes in the profession, as there are in the Church and the law. It is said that one or two of our leading consultants make as much as twenty thousand a-year. The statement is open to grave doubt. The probability is that the incomes of the most eminent half-dozen British physicians and surgeons which could be selected do not average more than six or seven thousand a-year. "And very fine incomes too," says the landed proprietor, whose own rent-roll has been reduced from six or seven thousand a-year to less than half that amount. Most true; but first-rate physicians and surgeons are to be numbered in Great Britain by the hundred, not by the halfdozen, and many of these are thankful to scrape along in expensive houses and costly social surroundings, with incomes that range between a thousand and fifteen hundred a-year. Not a few doctors of the first eminence can hardly make ends meet. In London many of them are said to be bound hand and foot to the money-lender. This would seem quite probable; for numbers of medical men, the present writer included, receive circulars from money-lenders, with offers of unlimited loans, every few weeks. A great many physicians and surgeons who have begun as consultants, and struggled on with heart-breaking alternations of hope and despair for ten or fifteen years, have been glad to find havens of quiet rest in country villages as general practitioners.

Notwithstanding all these undoubted drawbacks, medicine is not without its allurements as a profession. To begin with, it is eminently an honest calling. Of course, there are plenty of knaves in it, because there are plenty of idle and incompetent men. And the idle and incompetent in every calling are practically under the necessity of being knavish. But in medicine itself, as practised by the thoroughly educated physicians and surgeons of the present day, there is no particular temptation to dishonesty. The temptations of the bar and of the Church are much more severe. The lawyer can hardly help taking sides, and constantly doing his utmost to make the worse appear the better reason. This necessity is often entirely destructive of the scientific habit of mind, and not seldom ruinous to the character. It will hardly be possible for a good many lawyers to escape final perdition.

Similarly the temptations to equivocation and want of candour are very great in the Church; that is to say, in all churches. It is reported that Cardinal Laplace, now Archbishop of Rennes, once said to Father Hyacinthe in conversation that "Bishops nowadays have a double tongue; they are compelled to say aloud the very contrary of what they think." There is no doubt that many of the preachers of all churches have to close their eyes and ears against much of the illuminating knowledge and criticism of the present time. They dare not think; and even what they do think and cannot help thinking, they are afraid to say aloud. Even in the Church of England, where the teacher's liberty is larger than in any other church, this is nevertheless true. It is true, and it is as demoralising as it is certain. Religion is beautiful and priceless; but theological dogmas that have outlived their natural term of existence are often as destructive to the intellect and the soul as material putrefactions are to the bodily organism. The doctor is happy in that he has no temptation to play the knave. When once he has established his character for professional competence and personal sincerity, he finds his patients willing and anxious to walk by his simple word.

Whatever medicine may have been in pre-scientific days, at the present time it is one of the most interesting of all callings to the trained and cultivated intellect. The doctor is a worker in the field of science. He goes down to the roots and beginnings of life; of life physical, life intellectual, life moral. He considers nothing foreign to him which appertains to man. He studies humanity in the individual and in the mass. He sets before himself the task of carrying his own particular patients safely and happily through all the perils and dangers of this mortal life, and of securing for them a contented and peaceful old age. He does more than this: he looks for arguments in the nature and history of the human species which shall justify a belief in such a metamorphosis as will secure psychical continuity, and an ever-increasing intellectual expansion and moral elevation. In a word, he strives to see reasons for the resurection of the dead and the hope of an immortal life. Medicine is no longer the mere art of healing; it aims at being the true and proper science of man. Those who are in it and who see its possibilities and its greatness hold out the right hand of welcome to all young men of intellect, conscience, and nobility of mind. The dull, the selfish, the money-loving, and the vicious are requested to betake themselves elsewhere.

